# *TMEM18* is a regulator of adipogenesis and involved in *PPARG* signalling *in vivo*

**DOI:** 10.1186/2194-7791-2-S1-A25

**Published:** 2015-07-01

**Authors:** Kathrin Landgraf, Julian Schwartze, Denise Rockstroh, Wieland Kiess, Antje Körner

**Affiliations:** Center for Pediatric Research Leipzig (CPL), Hospital for Children and Adolescents, University of Leipzig, Leipzig, Germany

## Meeting abstract

Polymorphisms in *TMEM18* are associated with obesity in children and adults. We have reported previously that *TMEM18* is a regulator of human adipogenesis *in vitro* [1]. The aim of this study was to investigate the role of *TMEM18* during adipose tissue (AT) accumulation *in vivo*. For this, we used the zebrafish as an *in vivo* model for AT development. In addition, we analysed *TMEM18* expression in whole AT samples, isolated adipocytes and cells of the stroma-vascular fraction (SVF) from lean and obese children of our Leipzig Childhood AT cohort [2], and addressed associations with *PPARG* expression and obesity-related parameters.

Using whole mount *in situ* hybridisation on 9 day old zebrafish larvae, we showed co-expression of *tmem18* and *pparg* in a visceral region where also first adipocytes are detectable by Nile red staining. Morpholino-mediated inhibition of *tmem18* expression resulted in a reduction in the number of visceral adipocytes but did not affect zebrafish development *per se*. There was no effect of *tmem18* knockdown on eating behaviour suggesting that the inhibition of adipocyte formation was not mediated by a central effect. *Tmem18*-mediated inhibition of adipogenesis was accompanied by a significant down-regulation in *pparg* expression. Using luciferase reporter assays in 3T3-L1 cells, we detected a significant activation of the *PPARG* promoter in presence of Tmem18 indicating that Tmem18 is an upstream regulator of *PPARG* signalling. In line with these data, *TMEM18* expression correlated with *PPARG* expression in whole AT and isolated adipocytes but not in SVF cells of children included in our Leipzig AT cohort. Similar to *PPARG, TMEM18* expression was down-regulated in adipocytes of obese children compared to lean children and correlated with adipocyte diameter, macrophage infiltration, serum adiponectin levels and HOMA-IR as a measure of insulin resistance.

Our findings may indicate a potential role of *TMEM18* as a regulator of *PPARG* signaling during adipogenesis *in vivo*.
